# 2584. Predictors of Clinically Significant Infection in Patients with *Mycobacterium simiae* Isolated from Respiratory Samples: a Retrospective Study from a Tertiary Care Center in Lebanon

**DOI:** 10.1093/ofid/ofad500.2199

**Published:** 2023-11-27

**Authors:** Johnny Zakhour, Saliba Wehbe, Souad Bou Harb, Mariam Chalhoub, Elio Bitar, Souha S Kanj

**Affiliations:** American University of Beirut Medical Center, Baabda, Mont-Liban, Lebanon; American University of Beirut Medical Center, Baabda, Mont-Liban, Lebanon; American University of Beirut Medical Center, Baabda, Mont-Liban, Lebanon; American University of Beirut Medical Center, Baabda, Mont-Liban, Lebanon; American University of Beirut Medical Center, Baabda, Mont-Liban, Lebanon; American University of Beirut Medical Center, Baabda, Mont-Liban, Lebanon

## Abstract

**Background:**

*Mycobacterium simiae* is a rare slow-growing, non-tuberculous mycobacterium (NTM). It is predominant in Middle Eastern countries like Israel, Iran and Lebanon and is the most isolated NTM spp. at our institution (40%). Little is known about its reservoirs, pathogenesis, risk factors and indications for treatment.

**Methods:**

We retrospectively studied patients with respiratory cultures showing *M. simiae* at the American University of Beirut Medical Center (AUBMC) over 19 years. We used the American Thoracic Society/Infectious Diseases Society of America (ATS/IDSA) criteria to define NTM lung disease. Samples were cultured at AUBMC using Lowenstein-Jensen media and the BacT/ALERT system. NTM isolates were referred for speciation and susceptibility testing at Mayo Clinic, MN, USA.

**Results:**

We identified 72 isolates from 66 patients. Most were obtained from sputum samples (57.6%), the rest from bronchoalveolar lavages (BAL). Acid-fast bacilli smear was positive in 27% of samples with no difference between sputum and BAL. The median age of patients was 64 years and 51.5% were females. Bronchiectasis (34.5%), chronic obstructive pulmonary disease (27.3%) and immunosuppression (33.3%) were common (Table 1). As per the ATS/IDSA criteria, 67.9% of patients had NTM lung disease. The most common symptoms were cough (83.3%) and sputum production (74.5%). All patients except 2 had abnormal findings on CT imaging like nodules (58.5%), tree in bud aspect (45.3%) and bronchiectasis (24.5%). Among those, 55.6% had abnormal CT findings preceding the *M. simiae* isolation. Bronchiectasis was more common in those who satisfied the ATS/IDSA criteria versus those who did not (38.9% vs 20.0%). Otherwise, we found no significant differences between both groups (Table 2). Treatment was given to 88.9% of patients who satisfied the NTM lung disease and 42.1% of those who did not (p< 0.001) with a median duration of 12 months. Of those with available follow-up, relapse was reported in 24% of cases.

Characteristics of patients with M. simiae isolated from respiratory samples.

**Figure:**
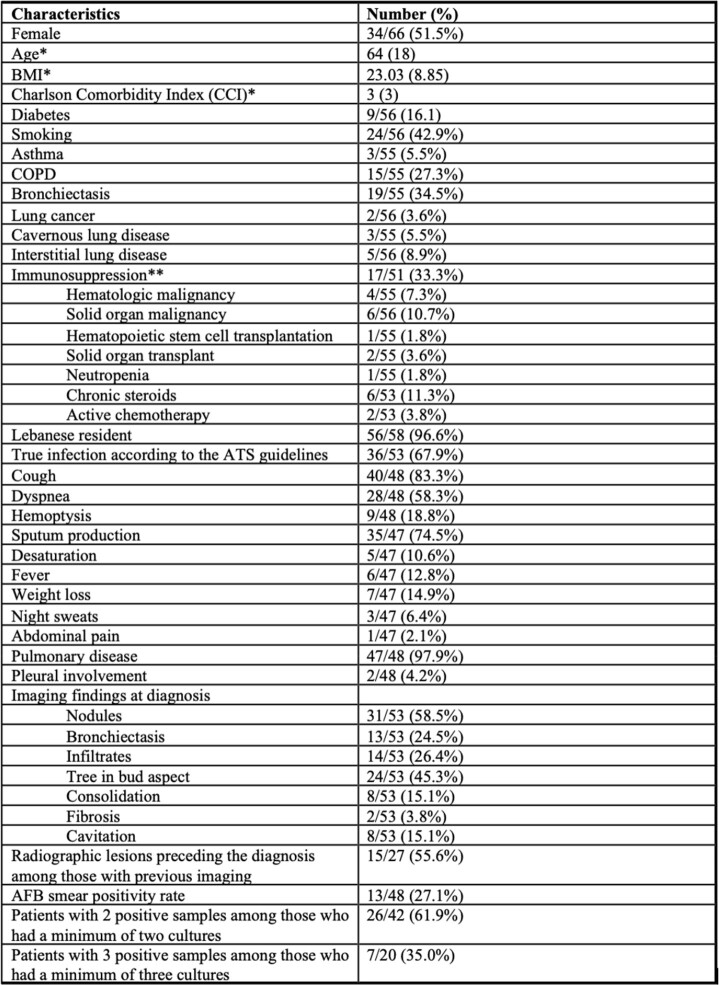

BMI, body mass index, COPD, chronic obstructive pulmonary disease, AFB, acid-fast bacilli. *Median(Interquartile range) **Composite variable including hematologic malignancy, solid organ malignancy, hematopoietic transplantation, solid organ transplant, chronic steroids treatment, and active chemotherapy.

Clinical and radiological differences between patients with M. simiae lung disease and those colonized according to the ATS/IDSA criteria.

**Figure:**
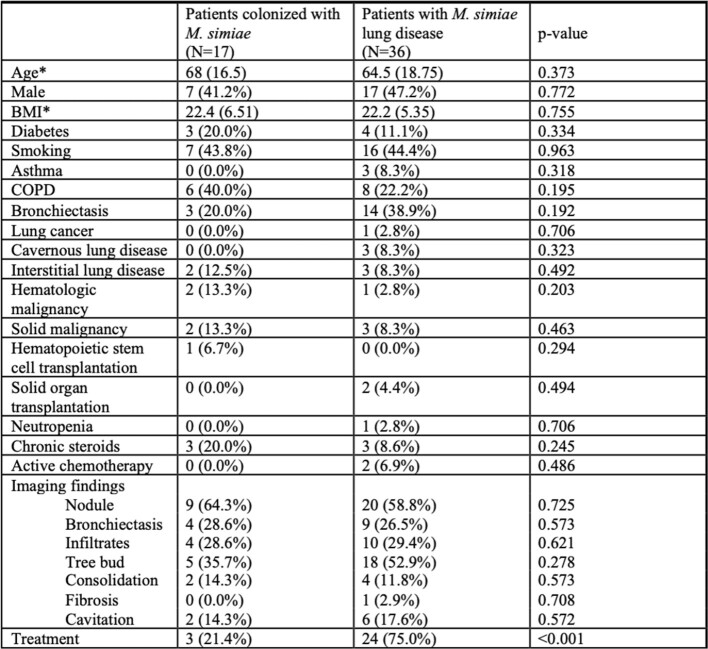

BMI, body mass index, COPD, chronic obstructive pulmonary disease. *Median (Interquartile range)

**Conclusion:**

*M. simiae* is a significant cause of NTM lung disease at our institution with a high rate of relapse. Further research is required to understand its geographic restriction, predisposing factors, and identify patients at risk for clinically significant infection.

**Disclosures:**

**Souha S Kanj, MD**, Gilead: Advisor/Consultant|Menarini: Advisor/Consultant|MSD: Advisor/Consultant|Pfizer: Advisor/Consultant

